# A transposable element annotation pipeline and expression analysis reveal potentially active elements in the microalga *Tisochrysis lutea*

**DOI:** 10.1186/s12864-018-4763-1

**Published:** 2018-05-22

**Authors:** Jérémy Berthelier, Nathalie Casse, Nicolas Daccord, Véronique Jamilloux, Bruno Saint-Jean, Grégory Carrier

**Affiliations:** 1 IFREMER, Physiology and Biotechnology of Algae Laboratory, rue de l’Ile d’Yeu, 44311 Nantes, France; 2 Mer Molécules Santé, EA 2160 IUML - FR 3473 CNRS, Le Mans University, Le Mans, France; 3 Institut de Recherche en Horticulture et Semences, INRA of Angers, AGROCAMPUS-Ouest, SFR4207 QUASAV, Université d’Angers, Angers, France; 4 Research Unit in Genomics-Info, INRA of Versailles, Versailles, France; 5 Université Bretagne Loire, Angers, France

**Keywords:** Transposable elements, Genome assembly, Pipeline, Tool, Annotation, Algae, Haptophyte, *Tisochrysis lutea*

## Abstract

**Background:**

Transposable elements (TEs) are mobile DNA sequences known as drivers of genome evolution. Their impacts have been widely studied in animals, plants and insects, but little is known about them in microalgae. In a previous study, we compared the genetic polymorphisms between strains of the haptophyte microalga *Tisochrysis lutea* and suggested the involvement of active autonomous TEs in their genome evolution.

**Results:**

To identify potentially autonomous TEs, we designed a pipeline named PiRATE (Pipeline to Retrieve and Annotate Transposable Elements, download: 10.17882/51795), and conducted an accurate TE annotation on a new genome assembly of *T. lutea*. PiRATE is composed of detection, classification and annotation steps. Its detection step combines multiple, existing analysis packages representing all major approaches for TE detection and its classification step was optimized for microalgal genomes. The efficiency of the detection and classification steps was evaluated with data on the model species *Arabidopsis thaliana.* PiRATE detected 81% of the TE families of *A. thaliana* and correctly classified 75% of them. We applied PiRATE to *T. lutea* genomic data and established that its genome contains 15.89% Class I and 4.95% Class II TEs. In these, 3.79 and 17.05% correspond to potentially autonomous and non-autonomous TEs, respectively. Annotation data was combined with transcriptomic and proteomic data to identify potentially active autonomous TEs. We identified 17 expressed TE families and, among these, a TIR/Mariner and a TIR/hAT family were able to synthesize their transposase. Both these TE families were among the three highest expressed genes in a previous transcriptomic study and are composed of highly similar copies throughout the genome of *T. lutea*. This sum of evidence reveals that both these TE families could be capable of transposing or triggering the transposition of potential related MITE elements.

**Conclusion:**

This manuscript provides an example of a de novo transposable element annotation of a non-model organism characterized by a fragmented genome assembly and belonging to a poorly studied phylum at genomic level. Integration of multi-omics data enabled the discovery of potential mobile TEs and opens the way for new discoveries on the role of these repeated elements in genomic evolution of microalgae.

**Electronic supplementary material:**

The online version of this article (10.1186/s12864-018-4763-1) contains supplementary material, which is available to authorized users.

## Background

Transposable Elements (TEs) are defined as DNA sequences able to move and spread within eukaryotic and prokaryotic genomes. These repeated elements constitute a variable fraction of eukaryotic genomes, ranging from 3% in the yeast *Saccharomyces cerevisiae*, 45% in human, to 80% in maize [1–3]. TEs were discovered by Barbara McClintock in the late 1940s, refuting the idea that genomes are stable but are, on the contrary, dynamic entities [4]. TEs are highly diverse and an unified classification system for eukaryotic TEs has been proposed, establishing two TE classes according to their transposition mechanisms, structures and similarities [5]. Class I (Retrotransposons) groups elements moving by a copy-paste mechanism through an RNA that is reversed transcribed. Class I is composed of several TE orders, named LTR, DIRS, PLE, LINE and SINE. Class II (DNA transposons) is composed of TEs using different cut-paste mechanisms to transpose. These elements are grouped into the orders TIR, Crypton, Helitron and Maverick. Although intact retrotransposons and DNA transposons are autonomous elements that can move by themselves, SINE elements are non-autonomous TEs and rely on LINE for their mobility, even though their origin is distinct. Other non-autonomous elements can also be distinguished. LTR elements can degenerate into non-coding structures known as LARD (> 4 kbp) or TRIM (< 4 kbp), and TIR elements can also degenerate into non-coding structures known as MITE. LARD, TRIM and MITE elements have intact termini and can thus move by exploiting the molecular machinery of related autonomous TEs [6]. Genomes also contain highly diverged TE fossils, accumulated over time and having no mobility capacity. Due to their mobility, TEs generate mutations in their host genome through new insertions/deletions and participate in genome evolution by impacting the DNA sequence, genome size [7, 8] and chromosome structure [9]. TE activity is known to be triggered during stressful events and, while the majority of transpositions are neutral or harmful to the organisms, transposition events are recognized to promote beneficial mutations [10]. New TE insertions can impact gene function and gene regulation [11]. They can also create new genes and participate in the rise of new phenotypes. The role of TEs has been widely studied in animals [12], land plants [13] and insects [14, 15], but work on their impact on microalgal genomes is only just beginning [16–19]. Microalgae form a diverse polyphyletic group composed of eukaryotic, unicellular and multicellular, photosynthetic organisms [20]. They live in all aquatic habitats whether these have fresh, brackish or salt water and have colonized different extreme habitats, ranging from hot springs, high altitude streams, ice sheets and desert sand crusts, highlighting their evolutionary ability to adapt to broad range of ecosystems [21–25]. Currently around 150,000 species of algae have been described (http://www.algaebase.org), but the number of non-described species is likely to number hundreds of thousands or millions of species [26]. They are divided among different eukaryotic phyla, in Archaeplastidia (green and red lineage), Rhizaria, Alveolates, Stramenopiles (brown lineage), Cryptophytes, Haptophytes and Excavates [27]. Despite their high number and diversity, few genome-wide TE annotations have been performed for microalgae. For the green lineage, this task was realized for ten Chlorophyte species [28–37]. For the red lineage, TE annotation was only done for the Rhodophyte *Cyanidioschyzon* sp. [38]. TEs were annotated in three diatom genomes (brown lineage) [18, 39–41] and also in five dinoflagellate species [42–46]. In Haptophytes, TE annotation has only been performed for one species [47]. These studies reveal that the TE content of microalgae genomes is diverse and includes both retrotransposons and DNA transposons.

Concerning TE activity in microalgae, a few studies have reported evidence of expression or transposition events. Expression of two LTR/Copia families was identified under nitrate starvation or exposure to diatom-derived reactive aldehydes in the diatom species *Thalassiosira pseudonana* and *Phaeodactylum tricornutum* [18]. Moreover, expression of LTR/Copia or TIR/Mariner elements was also reported under thermal stress in *P. tricornutum*, *Amphora acutiuscula*, *Amphora coffeaeformis* and *Symbiodinium microadriaticum* [16, 48–50]. Evidence of transposition events was only identified for a MITE element in a clone of *Chlamydomonas reinhardtii* in the presence of vitamin B_12_, resulting in a new phenotype [17].

Concerning TE activity in Haptophytes, we previously compared genetic polymorphisms between genomes of several strains of *Tisochrysis lutea* [51]. We identified new insertions/deletions and suggested the implication of autonomous TEs in the genome evolution of this species. In this context, the goal of the present study was to inventory TEs in the *T. lutea* genome and to identify potentially autonomous TEs. This marine microalga is commonly used as a feed in aquaculture [52] and is particularly studied for biotechnological applications such as food and biofuel production [53, 54]. In addition, several domesticated strains of *T. lutea* have been obtained with different processes [55] and a large amount of omics data has been collected [51, 56–60].

In this study, we present a detailed TE annotation of the *T. lutea* genome. To achieve this, we designed a new pipeline named PiRATE (Pipeline to Retrieve and Annotate Transposable Elements). The efficiency of the detection and classification steps of PiRATE was evaluated with data of the model species *Arabidopsis thaliana*. Moreover, to be as exhaustive as possible about the repeated content of *T. lutea*, a new genome assembly was performed by combining Pacific Bioscience and Illumina data. Finally, available transcriptomic and proteomic data were used to reveal potential active TE families.

## Results

### PiRATE: Pipeline to Retrieve and Annotate Transposable Elements of non-model organisms

The goal of the present study was to inventory the TE content of the *T. lutea* genome and study the activity of potentially autonomous TEs. Annotation of TEs is a challenging task because of their diversity, their repetitive nature and the complexity of their structures (i.e. GC-rich regions, homopolymers and repeated motifs). Numerous tools have been designed to identify TEs (Additional file 1: Table S1), which can be grouped into four approaches according to their TE detection method: (1) similarity-based detection such as RepeatMasker [61], (2) structure-based detection such as MITE-Hunter [62], (3) repetitiveness-based detection such as RepeatScout [63], and (4) tools building repeated elements from unassembled data such as dnaPipeTE [64].

**Fig. 1:**
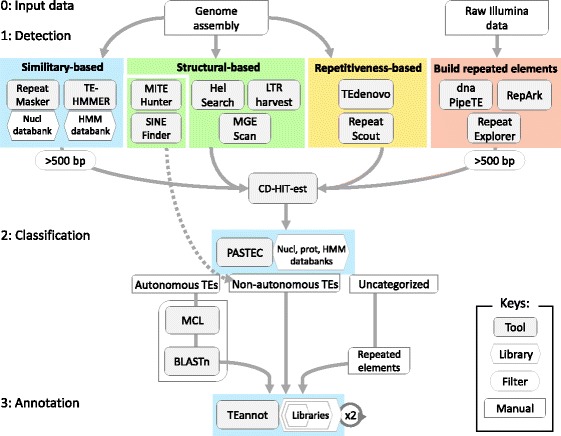
Overview of the PiRATE pipeline. Step 0: genome assembly and raw Illumina data are used as input data. Step 1: The detection of putative TEs and repeated sequences is performed using 12 tools, combining four detection approaches. Detected sequences from approaches 1 and 4 are filtered according to their length (minimum 500 bp). Detected sequences from the tools MITE-Hunter and SINE-Finder are directly saved as non-autonomous TEs. Other detected sequences are clustered with CD-HIT-est to reduce redundancy. Step 2: Putative TE sequences are automatically classified with PASTEC as potentially autonomous TEs, non-autonomous TEs or uncategorized sequences. The potentially autonomous TEs are manually checked and grouped into TE families. Step 3: Three libraries are manually constructed with a “Russian doll” strategy: 1) a “potentially autonomous TEs library”, a “total TEs library” and a “repeated elements library”. A double-run of TEannot is carried out for each library to select sequences that align with a full-length (FLC) on the genome assembly and finally obtain three independent annotations

Currently, the tool used most frequently to perform a TE annotation is RepeatMasker, which provides a rough estimation of the TE content in a genome assembly [61, 65]. However, this tool compares the genomic sequences with a databank of known TEs to realize the annotation and is therefore not suitable for realizing a de novo TE annotation [65–67]. To perform a de novo TE annotation, pipelines employing repetitiveness-based methods of detection, such as RepeatModeler and REPET, are commonly recommended [66–69]. Here we built PiRATE (Fig. 1) to conduct a de novo TE annotation in the genome of non-model species *T. lutea*. PiRATE is composed of detection, classification and annotation steps.

#### Detection of TEs

To date, genome assembly of non-model organisms has usually not been performed at the level of complete chromosomes but is instead highly fragmented. This fragmentation is recognized to be partly the result of a bad assembly of the TE copies due to their high repetitive content, which increases the difficulty of their detection [70]. The optimization of the detection step of PiRATE was therefore a priority. We made an overview of tools related to TE detection (Additional file 1: Table S1) and 12 tools were selected according to the specificity and efficiency of their algorithms. These tools represent the four major TE detection approaches (presented above), so as to be as exhaustive as possible. Combining tools is recognised to improve TE detection efficiency [66, 67, 71]. We then applied a clustering method to decrease the redundancy of the detected sequences, by selecting the larger detected sequences of each cluster. The goal of this step was to promote the detection of full-length TE sequences. The detection of complete TE sequences bearing recognizable conserved domains or specific structures and motifs makes the classification step easier. Moreover, a complete TE sequence indicates a potentially autonomous element.

#### Classification of TEs

The classification step of PiRATE is performed by PASTEC [72], which partly uses databanks of known TEs to establish an automated classification of the detected sequences. To improve the classification step of PiRATE, its default databanks were upgraded, by adding 1240 TE sequences from other public databanks, non-inventoried algal TEs and by building 78 new profile HMMs (Hidden Markov Model). Adding non-inventoried data is important for improving the TE classification of species belonging to poorly studied phyla, which often have few described TEs in the databanks. This is common for numerous microalgal phyla (i.e. Haptophyta, Euglenophyta and Dynophyta). In our case, only 17 TE families belonging to the Haptophyte phylum are present in the most frequently used and complete TE databank Repbase [73, 74]. We also estimated that only 2609 TE families are described for microalgal taxa in Repbase. Compared with other taxa, this number is very low, for examples 29,503 TE families are described for Metazoa and 12,620 for Viridiplantae (Repbase, 10/29/2017). The putative TE sequences are classified following the Wicker et al. classification [5] and can be grouped as 1) potentially autonomous TEs, 2) non-autonomous TEs or 3) uncategorized sequences. Because we were interested in potentially autonomous TEs, these sequences were manually checked and grouped into families.

#### Annotation of TEs

For the annotation step, we built three libraries in order to then apply a method that we named “Russian doll”, due to its nesting strategy (Additional file 1: Figure S1). We built a “potentially autonomous TEs library” containing checked potentially autonomous TEs, a “total TEs library” also containing the non-autonomous TEs, and a “repeated elements library” also containing the uncategorized repeated sequences. These nested libraries made it possible to perform several independent annotations in order to avoid a competition effect among sequences aligning on the same genomic regions.

### Evaluation of PiRATE with *A. thaliana* genomic data

#### Evaluation of the detection step

**Fig. 2:**
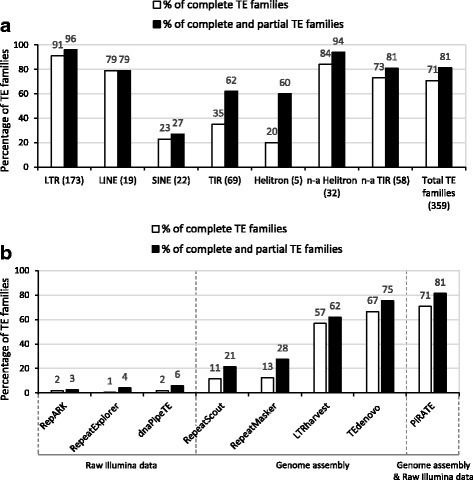
Evaluation of the detection step of PiRATE with genomic data of *Arabidopsis thaliana*. **a**) Percentage of TE families detected in *A. thaliana*. For each TE order (x-axis) is indicated the percentage of TE families detected with a complete length (coverage score ≥ 70%, white bars) or detected with a partial and a complete length (coverage score ≥ 40%, black bars). The x-axis indicates the number of TE families for each order; “n-a” means non-autonomous. **b**) Comparison of the percentage of TE families of *A. thaliana* detected by PiRATE (Step 1), RepARK, RepeatExplorer, dnaPipeTE, RepeatScout, RepeatMasker, LTRharvest and TEdenovo. For each tool is indicated the percentages of TE families of *A. thaliana* detected with a complete length (coverage score ≥ 70%, white bars) or detected with a partial and a complete length (coverage score ≥ 40%, black bars). The x-axis indicates the tools and nature of the input data

The detection and classification steps of PiRATE were evaluated to highlight their strengths and weaknesses. This evaluation made it possible to define suitable rules for the manual check step. As a control, we used 359 consensus sequences of the described TE families of *A. thaliana,* available in Repbase*.* Genomic data of the model plant *A. thaliana* provided a suitable control because of its high quality genome assembly and high TE diversity. Class I and Class II *A. thaliana* TE families are well described for both autonomous and non-autonomous TEs. Detected sequences covering less than 40% of the full-length of a consensus sequence were considered too short to be efficiently classified and were not taken into account. The proportion of TE families detected with a complete length (coverage score ≥ 70%) or detected with at least a partial length (coverage score ≥ 40%) is given in Fig. 2a. PiRATE detected ~ 81% (292/359) of the TE families described in *A. thaliana* genome (Fig. 2a)*.* PiRATE was especially effective for detecting sequences belonging to LTR (96%), LINE (79%), non-autonomous TIR (81%) and non-autonomous Helitron (94%) (Fig. 2a). It had a good efficiency for detecting TIR (62%) and Helitron (60%). However, it had difficulty detecting SINE elements (27%) (Fig. 2a). In addition, we compared the detection step of PiRATE to TEdenovo [68], LTRharvest [75], RepeatScout [63], RepeatMasker [61], dnaPipeTE [64], RepeatExplorer [76] and RepARK [77] (Fig. 2b). Overall, the detection step of PiRATE detected the highest percentage of TE families of *A. thaliana*. Compared to TEdenovo, which displayed the second highest percentage of detected TE families, PiRATE detected 21 additional TE families (+ 6%) (Fig. 2b and Additional file 1: Figure S2). PiRATE was particularly more effective for detecting LINE (+ 32%) and TIR (+ 10%) (Additional file 1: Figure S2).

#### Evaluation of the classification step

To evaluate the classification step of PiRATE, we used the 292 sequences detected by PiRATE during the evaluation of the detection step, which represent the largest detected sequences of the 292 TE families of *A. thaliana*. These 292 sequences were classified with PASTEC using the PiRATE databanks (excluding data from *Arabidopsis* species). To estimate the classification efficiency, we counted the number of detected TEs with correct classification at the order level and the number of sequences that had an incorrect classification or that were uncategorized. We observed that 75% (218/292) of the detected TEs were correctly classified, 7% (21/292) were incorrectly classified and 18% (53/292) were uncategorized. The classification step of PiRATE was therefore efficient at correctly classifying autonomous TEs belonging to LTR (98%), LINE (87%), TIR (91%) and Helitron (100%) but had difficulty correctly classifying SINE (50%), non-autonomous TIR (27%) and non-autonomous Helitron (7%) (Additional file 1: Figure S3). Taking into account all of the above results, PiRATE is efficient enough to detect and correctly classify the majority of the autonomous TE families.

### A new genome assembly of *T. lutea* to improve the TE annotation

We recently published a draft genome assembly of *T. lutea* obtained with Illumina short-read technology [51]. To obtain an improved genome assembly, the genome of *T. lutea* was re-sequenced with Pacific Bioscience long-read technology. A new genome assembly was performed from the long reads and was improved with the Illumina short-read data, used to build the draft genome assembly [51]. The new genome assembly of *T. lutea* is composed of 193 contigs and is 82 Mb in size. A gain of around 30 Mb was obtained (+ 34%), compared with the previous 54 Mb genome assembly, which was composed of 7659 contigs [51]. The size of the coding regions increased slightly between these genome versions. While the new genome assembly encodes for 15,972 genes, corresponding to a coding region length of 32 Mb, the gene proportion of the previous draft genome version was 25 Mb, suggesting that the new assembled regions are mostly repeated elements. This new larger version of the genome seems to incorporate more assembled TEs.

### Effect of genome quality on TE detection approaches

**Fig. 3:**
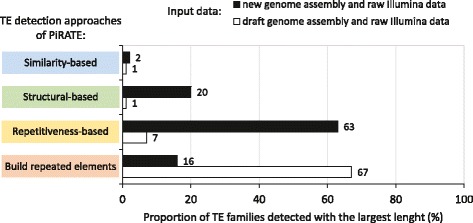
Comparison of the contribution of the four TE detection approaches of PiRATE on the detection of the TE families of *Tisochrysis lutea*, depending on the input data. For each TE detection approach, we calculated the number of TE families detected with the largest length and divide this number by the total of detected TE families of *T. lutea*. The input dataset was either the draft genome assembly of *T. lutea* and raw Illumina data of *T. lutea* (white bars) or the new genome assembly of *T. lutea* and raw Illumina data of *T. lutea* (black bars). The similarity-based detection, structural-based detection and the repetitiveness-based detection use a genome assembly as input data. The last approach builds repeated elements from raw Illumina data

To estimate the contribution of each TE detection approach of PiRATE depending on the level of fragmentation of the genome assembly, the detection step (Fig. 1) of PiRATE was applied with raw Illumina data of *T. lutea* and, either the draft genome version of *T. lutea* (7659 contigs) [51] or the new genome assembly of *T. lutea* (193 contigs). In both cases, the detected sequences were compared to the referent sequences of the TE families of *T. lutea* (described below). For each TE detection approach in PiRATE, we counted the number of *T. lutea* TE families detected, with the largest length (i.e. the most complete sequences, having the highest percentage of coverage compared to the reference TE sequences) and divide this number by the total of detected TE families. This provided an estimation ratio of the contribution of each TE detection approach depending on the input data (Fig. 3). With both types of dataset, the similarity-based approach had the weakest percentage and contributed to detecting only 2 or 3% of the *T. lutea* TE families. Using the draft genome assembly and the raw Illumina data, the structural-based approach contributed to detecting 1% of the TEs families of *T. lutea,* but 20% of the TE families of *T. lutea* with the new genome assembly and the raw Illumina data (Fig. 3). The repetitiveness-based approach contributed to detecting 7% of the TE families of *T. lutea* with the draft genome assembly and the raw Illumina data. However, it was the most efficient approach with the new genome and contributed to detecting 63% of the *T. lutea* TE families (Fig. 3). When a draft genome assembly is used as input, the fourth detection approach, using raw Illumina data to build repeated elements, was the most useful approach and contributed to detecting 67% of the TE families (Fig. 3).

### Annotation of the repeated elements content of the *T. lutea* genome

We applied PiRATE to the new genome assembly of *T. lutea* and raw Illumina data. After the classification step, we manually curated the sequences as potentially autonomous TEs, non-autonomous TEs or uncategorized repeated elements. Because we were interested in characterizing their activity, the potentially autonomous TEs were manually checked and grouped into families (see Methods). We identified six potentially autonomous families of LTR/Copia and four families of LTR/Gypsy (Table 1). We found 14 potentially autonomous families of LINE elements, similarly close to Tx1 elements, belonging to the L1 superfamily [78, 79]. We identified seven potentially autonomous families of TIR/Harbinger, six families of TIR/PiggyBac and eight families of TIR/Mariner. A high number of potentially autonomous hAT elements were detected. Due to their divergence, they were grouped into 129 putative families.

**Table 1:** Diversity and proportion of transposable element orders and classes in the genome assembly of *Tisochrysis lutea*. The abbreviations “a” and “n-a” indicate autonomous and non-autonomous transposable elements respectively

		Orders/ Superfamilies	Number of families (f) or detected sequences (s)	Number of potentially autonomous TEs	Proportion of the potentially autonomous TEs (%)	Proportion of total genome (%)
Class I	a	LTR/Copia	6 f	45	0.37	1.09
		LTR/Gypsy	4 f	242	2.56	4.65
		LINE/L1	14 f	59	0.25	3.87
	n-a	SINE	14 s			0.04
		LTR/LARD	17 s			0.76
		LTR/TRIM	240 s			5.48
Total Class I						15.89
Class II	a	TIR/hAT	129 f	145	0.41	2.12
		TIR/Mariner	8 f	41	0.11	0.19
		TIR/Harbinger	7 f	26	0.05	0.34
		TIR/PiggyBac	7 f	14	0.04	0.26
	n-a	MITE	188 s			2.04
Total Class II						4.95
Total TEs				572	3.79	20.84

Three annotations were conducted with three nested libraries (Additional file 1: Figure S1). From the “potentially autonomous TEs library” composed of 240 referent sequences, we estimated that the proportion of the potentially autonomous TEs represent 3.79% of the *T. lutea* genome (Table 1). The annotation of the TE content was performed with the “total TEs library” containing 459 supplementary sequences corresponding to 14 sequences of potential SINE elements, 188 sequences of potential MITE, 240 sequences of potential TRIM and 17 sequences of potential LARD (Table 1). From this annotation, we estimated that the genome of *T. lutea* contains 20.84% of potentially autonomous and non-autonomous TEs (Table 1 and Additional file 1: Figure S4). Class I and Class II TEs represent 15.89 and 4.95%, respectively (Table 1). We found a large quantity of Gypsy (4.65%), LINE (3.87%) and hAT (2.12%) copies, suggesting ancient burst events for these elements (Table 1). We established that the proportion of non-autonomous TEs is 17.05% (Table 1). Then, we performed the annotation of every repeated element by using the “repeated elements library” containing an additional 2680 uncategorized repeated sequences. From this annotation, we estimated that 17.79% of the *T. lutea* genome is represented by uncategorized repeated elements (Additional file 1: Figure S4). To estimate the proportion of the simple tandem repeats, we used the tool RepeatMasker and found that they made up 5.97% of the genome assembly of *T. lutea* (Additional file 1: Figure S4). By adding together the proportions of all the annotated repeats, we estimated that the total proportion of repeated elements in the *T. lutea* genome was 44.6%. Knowing that the coding gene proportion is of 38.49%, we estimated that 16.91% of the genome is non-characterized (Additional file 1: Figure S4).

### Discovery of potentially active autonomous TEs in the *T. lutea* genome

**Fig. 4:**
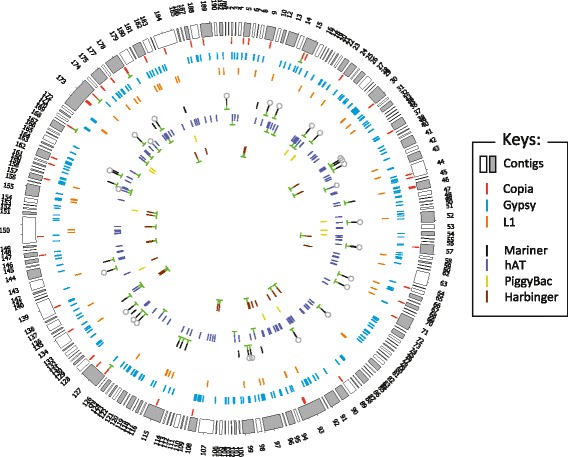
Cartography of the 572 potentially autonomous TEs in the genome assembly of *Tisochrysis lutea*. The contig position is random. TEs belonging to the same superfamily are represented with the same colour. The 73 potentially autonomous TEs belonging to the 17 expressed TE families are highlighted with a green “T”. Elements belonging to the TIR/Mariner *Luffy* family and TIR/hAT *Ace* family are marked by a grey circle. Transposase proteins were synthesized for these both TE families

In this study we chose to focus on the identification of potentially autonomous TEs to reveal potentially active elements. From the annotation obtained with the “potentially autonomous TEs library”, we performed the cartography of the 572 annotated TEs that are potentially autonomous (Fig. 4).

To identify potentially active TEs and have an estimation of the genome dynamic of *T. lutea*, transcriptomic data were mapped on the new genome assembly and crossed with the annotation of the 572 potentially autonomous TEs. Expression was identified for 17 TE families: one LTR/Copia, four TIR/Mariner, four TIR/Harbinger and eight TIR/hAT. These families represent 73 potentially autonomous TEs and their genomic position is illustrated in Fig. 4 and is indicated in Additional file 2. Putative ancient transpositions were studied by looking for similarities between copies belonging to these 17 expressed TE families (Additional file 2). We identified that the Mariner-3 family is composed of 24 highly similar copies, which share a mean pairwise identity of 99.7%. Among them, 20 copies seem to be complete (Additional file 3). This high number of similar copies suggests that this family was/is active. The hAT-2 family is composed of three highly similar copies that share a mean pairwise identity of 99.8%. Moreover, eight similar copies were identified for the Harbinger-6 family and five similar copies for the Copia-3 family. Other details can be found in Additional file 2. TE copies belonging to these 17 expressed TE families were submitted to BLASTx on proteomic data of *T. lutea*, that we previously obtained under nitrogen limitation [58]. We identified that transposase proteins were synthesized for the Mariner-3 family and the hAT-2 family. The transposases of the Mariner-3 and hAT-2 families match with six and 36 peptides, respectively. The alignments with the matching peptides can be found for both families in Additional file 4. From transcriptomic data of a previous study, we highlight that these families were among the three higher expressed genes [58].

## Discussion

### PiRATE: Pipeline to Retrieve and Annotate Transposable Elements of non-model organisms

The goal of the present study was to inventory the TE content in the genome of *T. lutea* genome and study the activity of potentially autonomous TEs. We built PiRATE to counter the lack of knowledge about TEs in Haptophytes and the difficulty of identifying TEs in a fragmented genome assembly [70]. The detection step of PiRATE has been optimized to promote the detection of full-length TE sequences and its classification step has been improved for algal genomes. The detection step of PiRATE combines multiple, existing analysis packages representing all major approaches for TE detection. The detection step of PiRATE was evaluated with genomic data of *A. thaliana* and compared to TEdenovo [68], LTRharvest [75], RepeatScout [63], RepeatMasker [61], dnaPipeTE [64], RepeatExplorer [76] and RepARK [77] (Fig. 2b). Overall, the detection step of PiRATE detected the highest percentage of TE families (81%) with a partial and complete length compare to the other tools used alone (Fig. 2b). This confirms that the combining of multiple tools, using several approaches improves the detection of different TE families, with complete sequences, as previously indicated [66, 67, 71]. In this comparison, TEdenovo was efficient and displayed the second highest percentage of detected TE families (75%) (Fig. 2b). LTRharvest also showed a good capacity to detect TE families of *A. thaliana* (62%) (Fig. 2b). This is due to the high content of LTR elements in the *A. thaliana* genome and because this tool detected TE families belonging to other TE orders. In this comparison, the least effective tools were RepARK (3%), RepeatExplorer (4%) and dnaPipeTE (6%), which used raw illumina data as input (Fig. 2b). This is not surprising considering the challenge of building repeated elements from raw Illumina data, compared to the other tools using the complete genome assembly of *A. thaliana*.

### A new genome assembly of *T. lutea* to improve the TE annotation

We recently published a draft genome assembly of *T. lutea* obtained with Illumina short-read technology [51]. While this technology has a very low sequencing error rate, its use alone often leads to fragmented assemblies, especially in TE-rich genomes, due to the incapacity of short-reads to entirely span repetitive elements [80]. To obtain an improved genome assembly, the genome of *T. lutea* was re-sequenced with Pacific Bioscience long-read technology and the assembly was corrected with short-read Illumina data. Indeed, the use of long-reads leads to a more complete and accurate assembly of long repeated elements such as TEs [81–83]. However, to date, this technology has a high sequencing error rate and its combination with short-read Illumina data has become a common way of partially overcoming this problem [84–86]. Compare to the previous draft genome assembly, this new genome assembly is larger, less fragmented and seems to incorporate more assembled TEs.

### Effect of genome quality on TE detection approaches

To estimate the contribution of the four TE detection approaches of PiRATE depending on the level of fragmentation of the genome assembly, the detection step (Fig. 1) of PiRATE was applied with raw Illumina data of *T. lutea* and, either the draft genome version of *T. lutea* (7659 contigs) [51] or the new genome assembly of *T. lutea* (193 contigs). The four TE detection approaches showed different contribution according to the level of fragmentation of the genome assembly (Fig. 3). By gathering these four detection approaches, PiRATE improves the TE detection of organisms having a genome assembly which is highly fragmented.

### Annotation of the repeated elements content of the *T. lutea* genome

With PiRATE, we established that the total proportion of repeated elements in the *T. lutea* genome is represented by 20.84% of TEs, 17.79% of uncategorized repeated elements and 5.97% of simple tandem repeats (Additional file 1: Figure S4). The high percentage of uncategorized repeated elements could indicate the presence of unknown TEs. A high number of uncategorized sequences (30.9%) was also reported in the *Emiliania huxleyi* genome [40]. Here, we choose to focus on the identification of potentially autonomous TEs to reveal potentially active elements. The proportion of the potentially autonomous TEs represents 3.79% of the *T. lutea* genome, corresponding to 572 annotated TEs (Fig. 4). Interestingly, we found a potentially autonomous TIR/Mariner in the predicted mitochondrial genome and a potentially autonomous LTR/Copia and TIR/hAT in the predicted chloroplast genome.

### Identification of potentially active TEs in *T. lutea*

Few studies have investigated TE activity in microalgal genomes and their role is poorly known. Regarding Class I TEs, some studies reported expression of LTR elements in dinoflagellate and diatom species under thermal stress or nitrogen limitation [16, 48–50]. Concerning Class II elements, a previous study reported a case of phenotypic evolution for the microalga *Chlamydomonas reinhardtii* caused by the transposition of a MITE in the presence of vitamin B_12_ [17]. In the present study, we identified 17 expressed TE families and, among these, a TIR/Mariner *Luffy* and a TIR/hAT *Ace* family were able to synthesize their transposase under nitrogen starvation [58]. We highlight the presence of highly similar copies (Additional file 3) suggesting that these elements are able to transpose or could be able to trigger the transposition of potential derived MITE elements. Although we cannot draw conclusions about their mobility, the investigation of the TE expression is a good indicator of the potential activity of TEs. Nitrogen limitation has been previously described as a stress condition in the diatom *Phaeodactylum tricornutum*, triggering overexpression of the LTR/Copia family named *Blackbeard* [18]. Although we cannot draw conclusions about de novo insertions, the evidence presented here indicates that these both TEs families are suitable candidates for mobility and could participate in the genome evolution of *T. lutea*.

## Conclusion

Genome-wide TE annotation has rarely been performed in microalgae compared with animals, insects and land plants. This study opens the way to new searches about the role of TEs in the genome evolution of *Tisochrysis lutea* and their contribution to the microalgal adaptation process. In the present study, we built PiRATE to counter the lack of knowledge about TEs in Haptophytes and the difficulty of identifying TEs in a fragmented genome assembly. With PiRATE, we conducted a genome-wide detection and annotation of the repeated elements in a new genome assembly of *Tisochrysis lutea* and established that it is composed of 3.8 and 15.95% of potentially autonomous and non-autonomous TEs, respectively. The annotation of the potentially autonomous TEs was crossed with transcriptomic and proteomic data and evidence of expression was identified for 17 TE families. Among these, we discovered that transposase proteins were synthesized for both a Mariner (*Luffy*) and a hAT (*Ace*) family. Both these families have several highly similar copies throughout the genome and were among the three highest expressed genes in a previous transcriptomic study. All of this suggests that both these families could be able to transpose themselves or trigger the transposition of potential derived MITE elements.

## Methods

### Microalga strain and culture conditions

The *T. lutea* strain was provided by the Culture Collection of Algae and Protozoa (CCAP 927/14). This strain was isolated by Haines in the late 70s and stored in the algae bank. The strain was grown in two 1-L flasks, bubbled with 0.22 mm filtered-air. The culture was maintained at a constant temperature of 21 °C, under a constant irradiance of 50 μmol m^− 2^ s^− 1^.

### DNA extraction, sequencing, genome assembly and gene annotation

Total DNA was extracted from the *T. lutea* WT-strain using a phenol/chloroform protocol. DNA quality and concentration were assessed with gel electrophoresis and Qubit Fluorometric Quantitation (ThermoFisher, Massachusetts, USA), respectively. *T. lutea* genome sequencing was performed with a PacBio RSII sequencer (Pacific Bioscience, California, USA) at the Plateforme GeT PlaGe (Toulouse, France); seven SMRT cells were performed. Filtered subreads were assembled using Canu1.3 [82]. The assembly was polished with Quiver (https://github.com/PacificBiosciences/GenomicConsensus) and its accuracy was improved with Pilon [87] using previous Illumina Hiseq mate-pair reads of *T. lutea* ([51]; SRA: SRR3156597). The annotation of the coding-gene region was performed with the pipeline MAKER2 [88–91].

### TE annotation in the *T. lutea* genome using PiRATE

#### Step 1: TE detection

The new genome assembly of *T. lutea* and previous raw Illumina data ([51]; SRA: SRR3156597) were used as input. Putative TE sequences were detected using four approaches (Fig. 1). The first approach was represented by two tools using similarity-based detection: RepeatMasker (setting: -s, −no_low, −lib; with the PiRATE nucleotide databank; [61]) and TE-HMMER (with a homemade profile HMMs databank). TE-HMMER is a homemade tool using HMMER (default setting, [92]) and tBLASTn (setting: -evalue 10E-300, [93]). The second approach consisted of five tools using structural-based detection: LTRharvest (default setting, [75]), Helsearch (default setting, [94]), MGEScan-nonLTR (default setting, [95]), MITE-Hunter (default setting, [62]) and SINE-Finder (default setting, [96]). The third approach combines tools using repetitiveness-based detection: TEdenovo (steps 1 to 4, default setting, [68] and Repeat Scout (default setting, [63]). These tools cluster repeated sequences from a genome assembly to build consensus sequences. The last approach was composed of tools performing the assembly of repeated sequences from raw Illumina data (fasta or fastq). We used RepARK (default setting, [77]), dnaPipeTE (setting: %coverage: 0.6, [64]) and RepeatExplorer (setting: -paired, [76]). The sequences detected by the first and the last approaches that were below 500 bp in length were removed with a perl script. The sequences detected with SINE-Finder and MITE-Hunter were directly saved for the second step. Other detected sequences were concatenated into a single FASTA file and clustered with CD-HIT-est (settings: -aS 1 -c 1 -r 1 -g 1 -p 0, [97]) to reduce the redundancy. This made it possible to remove shorter sequences that aligned with 100% of identity on a part of the larger sequences.

#### Step 2: TE classification

In the second step, sequences were automatically classified with PASTEC [72], following the Wicker et al. classification system [5] This tool was improved with custom databanks (described below). Three libraries were manually constructed with a “Russian doll” strategy in order to perform separate annotations (Additional file 1: Figure S1): a “potentially autonomous TEs library”, a “total TEs library” containing the potentially autonomous TEs and the non-autonomous TEs and a “repeated elements library” also containing the uncategorized repeated sequences. Sequences classified as LTR, LINE and TIR were manually sorted by superfamily (according to the evidence section produced by PASTEC). To facilitate their manual check, sequences belonging to the same putative superfamily were grouped into families with MCL (MCL_inflation: 1.5; MCL_coverage: 0). The percentage of identity between sequences belonging to the same family were checked with Blastn (−identity: 80%). We followed the 80–80-80 Wicker rules to form families [5]. Finally, larger sequences from each TE family were checked and selected for the “potentially autonomous TEs library” according to the presence of TE domains or similarities with Pfam (http://pfam.xfam.org), NCBI-BLASTx and Censor (http://www.girinst.org/censor). We defined as potentially autonomous LTR, sequences bearing at least a reverse transcriptase and an integrase domain and having similarity to known LTR elements. We defined as potentially autonomous LINE, sequences bearing at least a reverse transcriptase domain and sharing similarity to known LINE elements. We defined as potentially autonomous TIR, sequences with evidence of a transposase domain or similarity with known TIR elements.

No manual checks were performed for sequences classified as non-autonomous TEs. Sequences classified as SINE, MITE and TRIM were directly selected for the “total TEs library”. Only sequences classified as LARD, which were obtained with the repetitiveness-based approach of TE detections (TEdenovo or Repeat Scout), were selected. Sequences detected by SINE-Finder and MITE-Hunter were also directly selected for the “total TEs library”. Finally, the sequences classified as noCat (uncategorized) and obtained with the repetitiveness-based approach at the TE detection step were selected for the “repeated elements library”.

#### Step 3: TE annotation

Three libraries were built (Additional file 1: Figure S1): 1) a “potentially autonomous TEs library” 2) a “total TEs library” and 3) a “repeated elements library”. A first run of TEannot ([68], default setting, steps 1, 2, 3, 7 and 8) was performed for each library to known sequences matching with a full-length size on the genome (FLC sequences) and to remove potential chimeric data. A second run of TEannot was performed with these FLC sequences for each of the final libraries (default setting, steps 1, 2, 3, 4, 5, 7 and 8) and three annotations were obtained.

### Proportion of TEs and repeated elements in *T. lutea*

From the annotation file obtained with the “potentially autonomous TEs library”, we manually selected 572 sequences and calculated their proportion in the genome of *T. lutea.* TEs. The different criteria used are detailed in Additional file 1: Method S1 and Table S2. An illustration of the position of these sequences on the *T. lutea* genome assembly was built with the tool Circos [98]. The annotations obtained with the “total TEs library” and the “repeated elements library” were used to estimate the total proportion of TEs and to calculate the proportion of uncategorized repeated elements in the genome of *T. lutea*. Details on the method are available in Additional file 1: Method S2 and Table S3. The proportion of simple repeats was calculated with the tool RepeatMasker (setting: -s -noint -no_is, [61]).

### PiRATE databanks

#### Nucleotide and protein databanks

A nucleotide and a protein databank of TEs were built with sequences from Repbase (REPET version 20.05, http://www.girinst.org/repbase), the P-MITE database (http://pmite.hzau.edu.cn) and SINE base (http://sines.eimb.ru). Because algae originally arose from the predation of a cyanobacterial organism by a eukaryotic heterotrophic organism, cyanobacterial TE sequences were also added from the IS-finder database (http://www-is.biotoul.fr) (Additional file 5). Moreover, we added non-inventoried TEs of microalgae and macroalgae, retrieved from the NCBI database (Additional file 5).

#### Profile HMMs databank

A homemade databank of profile HMMs was built with sequences of the protein databank. Multiple protein alignments were performed with Clustal Omega (http://www.ebi.ac.uk/Tools/msa/clustalo/). When possible, TE protein sequences from algae were favoured. 78 profile HMMs were performed with the HMMbuild tool of HMMER [92] for 62 TE categories displayed on the Browse Repbase tool (http://www.girinst.org/repbase/update/browse.php). This databank was used with TE-HMMER at the detection step. At the classification step, we combine this databank with the default databank of PASTEC (ProfilesBankForREPET_Pfam27.0_GypsyDB.hmm, https://urgi.versailles.inra.fr/download/repet).

### Evaluation of PiRATE

The efficiency of the detection and classification steps of PiRATE were evaluated with genomic data of the model plant *A. thaliana*. We used the genome assembly TAIR10 available on the TAIR project (https://www.arabidopsis.org/download/index-auto.jsp?dir=%2Fdownload_files%2FGenes%2FTAIR10_genome_release%2FTAIR10_chromosome_files) and the raw Illumina data available at the 1001 genome project http://1001genomes.org/data/SLU/SLUHenning2014/releases/current/strains/Seattle-0). These data of *A. thaliana* were submitted to the step 1 of PiRATE (RepeatMasker and TE-HMMER were used without data from *Arabidopsis* species in the databanks). The detected sequences were submitted to PASTEC [72] and compared to the 359 TE families described in *A. thaliana* and available on Repbase (http://www.girinst.org/repbase). We didn’t include the terminal repeated sequences of the LTR TE families and the heterologous TE named DRL1. From the classification file, we selected each of the sequences matching to a TE consensus sequences of *A. thaliana*. Those covering less than 40% of the full-length of a consensus sequences were considered as too short to be efficiently classified and were not taken in account. We considered as a partial or complete detection the detected sequences covering at least 40% or 70% of the full-length of a consensus TE family of *A. thaliana*, respectively*.* For the comparison of the detection step of PiRATE with TEdenovo [68] (steps 1 to 4, with LTRharvest [75]), LTRharvest [75], RepeatScout [63], RepeatMasker [61], dnaPipeTE [64], RepeatExplorer [76] and RepARK [77], the number of detected TE families was calculated with the same method previously described for the evaluation of the PiRATE detection step. For the evaluation of the classification step of PiRATE, we used the longest detected sequences of the 292 TE families detected by PiRATE during the evaluation of the detection step as a control. These 292 sequences were classified with PASTEC using modified versions of the three PiRATE databanks (Nucleotide, protein and profile HMMs), without data from *Arabidopsis* sp. We calculated the percentages of correct classification, incorrect classification or uncategorized classification. Details on the impact of the genome assembly quality on the efficiency of the TE detection step of PiRATE are available in Additional file 1: Method S3.

### Transcriptomic and proteomic analyses

The expression analysis was performed using eight sets of previously published transcriptomics data [56, 58]. These data were concatenated and normalized using the tool insilico_read_normalization.pl of Trinity [99]. Reads were then mapped on the new genome assembly of *T. lutea* with TopHat [100] and crossed with the annotation of the potentially autonomous TEs. HTseqCount [101] was used to count the number of mapped reads for each potentially autonomous TEs. With a homemade script we retrieved the TE families with transcripts covering at least 90% of the annotated sequences. Sequences of the TE copies of these expressed TE families were then compared with BLASTx to published proteomic data [58]. Sequence alignments of the peptides of the Mariner (*Luffy*) and hAT (*Ace*) elements on the predicted transposases were performed with ClustalOmega and visualized with Geneious (Additional file 4). With the global-alignment tool of Geneious [102] (setting: free end gaps), a mean pairwise identity was calculated for each expressed TE family having at least three annotated copies (Additional file 3).

### PiRATE is automated through a stand alone Galaxy

All tools used in PiRATE are automated in a standalone Galaxy [103]. The PiRATE-Galaxy is available through a virtual machine at 10.17882/51795. A tutorial file can be download.

## Additional files

Additional file 1: Additional supporting information. This file contains the additional supporting figures, tables, results and materials and methods. (PDF 594 kb)

Additional file 2: Percentage of identity between copies of the expressed TE families. This file lists the percentage of identity between the TE copies of the 17 expressed TE families identified in the genome of *Tisochrysis lutea*. (XLSX 19 kb)

Additional file 3: Sequences alignment of TE copies of the TIR/Mariner *Luffy* family. This file contains the sequence alignment of the copies belonging to the TIR/Mariner *Luffy* described in the genome of *Tisochrysis lutea*. (PDF 17427 kb)

Additional file 4: Sequences alignment of the peptides matching on the predicted TE proteins. This file contains the alignment of the peptides matching on the predicted proteins of the TIR/Mariner *Luffy* and the TIR/hAT *Ace*. (PDF 996 kb)

Additional file 5: List of non-inventoried sequences added to the databanks used by the pipeline PiRATE. This file lists the non-inventoried sequences added to the databanks used by the pipeline PiRATE, they belong to algae and cyanobacteria. (XLSX 23 kb)

## Abbreviations

HMM: Hidden Markov Model
LARD: Large Retrotransposon Derivative
LINE: Long Interspersed Element
LTR: Long Terminal Repeat
MITE: Miniature Inverted-repeat Transposable Element
PiRATE: Pipeline to Retrieve and Annotate Transposable Element
PLE: Penelope
SINE: Short Interspersed Nuclear Element
TE: Transposable element
TIR: Terminal Inverted Repeat
TRIM: Terminal-repeat Retrotransposons In Miniature
