# Supplementary material for: A transposable element annotation pipeline and expression analysis reveal potentially active elements in the microalga Tisochrysis lutea
Source: BMC Genomics. 2018 May 22;19:378. doi: 10.1186/s12864-018-4763-1 (PMC5963040; doi:10.1186/s12864-018-4763-1)
Supplement: Supplementary file 1 — Additional supporting information. This file contains the additional supporting figures, tables, results and materials and methods. (PDF 594 kb) [file 12864_2018_4763_MOESM1_ESM.pdf]

Additional file 1:

A transposable elements annotation pipeline and expression analysis reveal potentially active elements in the microalga *Tisochrysis lutea*

Jérémy Berthelier[1]*, Nathalie Casse[2], Nicolas Daccord[3], Véronique Jamilloux[4], Bruno Saint-Jean[1], Grégory Carrier[1]

1: IFREMER, Physiology and Biotechnology of Algae Laboratory, rue de l'Ile d'Yeu, 44311 Nantes, France.

2: Mer Molécules Santé, EA 2160 IUML, FR 3473 CNRS, Le Mans University, Le Mans, France

3: Institut de Recherche en Horticulture et Semences, INRA of Angers, France

4: Research Unit in Genomics-Info, INRA of Versailles, Versaille, France

*Corresponding author: berthelier.j@laposte.net

Table of Contents

Table S1: Bibliography of tools related to TE detection. 2

Figure S1: Illustration of the "Russian doll" strategy 3

Figure S2: Comparison of the detection step of PiRATE 4

Figure S3: Evaluation of the classification step of PiRATE. 5

Figure S4: Genome composition of *Tisochrysis lutea* 5

Method S1: Annotation of the potential autonomous TEs 6

Table S2: Rules established to annotate potentially autonomous TEs 6

Method S2: Annotation of TEs and repeated elements in the genome of *Tisochrysis lutea*. 7

Table S3: Rules established to annotate the TE content in the *Tisochrysis lutea* genome 7

Method S3: Contribution of each TE detection approach, depending on the input data. 8

Table S1: Bibliography of tools related to TE detection. The "*" indicates the 12 tools used in the detection step of PiRATE.

Name	Website	Author(s)
**Similarity-based tools**		
RepeatMasker*	www.repeatmasker.org/	Smit and Hubley, 1997
Censor	http://www.girinst.org/censor/download.php	Kohany et al., 2006
Windowmasker	ftp.ncbi.nlm.nih.gov/pub/agarwala/windowmasker	Morgulis et al., 2006
Transposon-PSI	transposonpsi.sourceforge.net/	Haas, 2007
TeClass	http://www.compgen.uni-muenster.de/teclass/index.hbi?	Abrusan et al., 2009
RepClass	https://sourceforge.net/projects/repclass/	Feschotte et al.,2009
TARGeT	http://target.iplantcollaborative.org	Han et al., 2009
TESeeker	http://repository.library.nd.edu/view/27/teseeker	Kennedy et al., 2011
PASTEClassifier	https://urgi.versailles.inra.fr/Tools/PASTEClassifier	Hoede et al., 2014
TE-HMMER*	http://www.seanoe.org/data/00406/51795/	Berthelier et al., 2017
**Structural-based tools**		
TRANSPO	http://alggen.lsi.upc.es/recerca/search/transpo/transpo.html	Santiago et al., 2002
TSDfinder	http://www.ncbi.nlm.nih.gov/CBBresearch/Landsman/TSDfinder/	Szak et al., 2002
LTR_STRUC	http://www.mcdonaldlab.biology.gatech.edu/ltr_struc.htm	McCarthy and McDonald, 2003
MAK	http://nar.oxfordjournals.org/content/31/13/3659.long	Yang and Hall, 2003
LTR_MINER	http://genomebiology.com/content/supplementary/gb-2004-5-10-r79-s5.pl	Pereira, 2004
LTR_par	http://www.eecs.wsu.edu/~ananth/software.htm	Kalyanaraman and Aluru, 2006
LTR_FINDER	http://tlife.fudan.edu.cn/ltr_finder/	Xu and Wang, 2007
LTRharvest*	http://www.zbh.uni-hamburg.de/?id=206	Ellinghaus et al., 2008
LTRdigest	http://www.zbh.uni-hamburg.de/?id=207	Steinbiss et al., 2009
MUST	http://www.healthinformaticslab.org/supp/	Chen et al., 2009
HelSearch*	http://omictools.com/helsearch-tool	Lixing and Bennetzen, 2009
MGEScan-LTR	http://darwin.informatics.indiana.edu/cgi-bin/evolution/daphnia_ltr.pl	H. Tang, 2009
MGEScan-nonLTR*	http://darwin.informatics.indiana.edu/cgi-bin/evolution/nonltr/nonltr.pl	H. Tang, 2009
MITE-Hunter*	http://target.iplantcollaborative.org/mite_hunter.html	Han and Wessler, 2010
Sine-Finder*	http://www.jstor.org/stable/41434686?seq=1#page_scan_tab_contents	Wenke et al., 2011
RSPB	http://pmite.hzau.edu.cn/MITE/tools/	Lu et al., 2012
MITE-Digger	http://omictools.com/mite-digger-tool	Yang, 2013
TIRfinder	https://sourceforge.net/projects/tirfinder/	Gambin et al., 2013
HelitronScanner	http://omictools.com/helitronscanner-tool	Xiong et al., 2014
detectMite	https://sourceforge.net/projects/detectmite/	congtig et al., 2016
SINE_scan	https://github.com/maohlzj/SINE_Scan	Mao and Wang, 2016
MUSTv2	http://www.healthinformaticslab.org/supp/resources.php	Ge et al., 2017
**Repetitiveness-based tools**		
REPuter	https://bibiserv2.cebitec.uni-bielefeld.de/reputer	Kurtz and Schleiermacher, 1999
RepeatFinder	http://cbcb.umd.edu/software/RepeatFinder/	Volfovsky et al., 2001

RECON	http://selab.janelia.org/recon.html	Bao and Eddy, 2002
GROUPER	https://urgi.versailles.inra.fr/Tools/REPET	Quesneville et al., 2003
PILER	http://www.drive5.com/piler/	Edgar and Myers, 2005
RepeatScout*	http://www.repeatscout.bioprojects.org/	Price et al., 2005
Repseek	http://wwwabi.snv.jussieu.fr/public/RepSeek/	Achaz et al., 2006
Pclouds	http://www.evolutionarygenomics.com/ProgramsData/PClouds/PClouds.html	Gu et al., 2008
Tallymer	http://www.zbh.uni-hamburg.de/?id=211	Kurtz et al., 2009
TEdenovo*	http://urgi.versailles.inra.fr/Tools/REPET	Flutre et al., 2011
RepeatModeler	http://www.repeatmasker.org/RepeatModeler.html	Smit and Hubley, 2014
**Build repeated elements**		
ReAS	ftp://ftp.genomics.org.cn/pub/ReAS/software/	Li et al., 2005
RepeatExplorer*	http://galaxy.umbr.cas.cz:8080/	Novák et al., 2013
RepARK*	https://github.com/PhKoch/RepARK	Koch et al., 2014
Tedna	https://urgi.versailles.inra.fr/Tools/Tedna	Zytnicki, 2014
dnaPipeTE*	https://lbbe.univ-lyon1.fr/-dnaPipeTE-	Goubert et al., 2015
Transposome	https://github.com/sestaton/Transposome	Staton and Burke, 2015
REPdenovo	https://github.com/Reedwarbler/REPdenovo	Chu et al., 2016

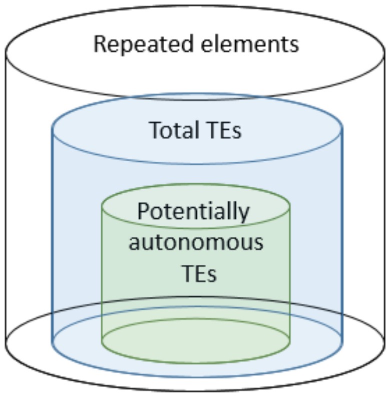

Figure S1: Illustration of the "Russian doll" strategy employed for the implementation of the libraries used to annotate the potentially autonomous TEs, total TE content and repeated element content of *Tisochrysis lutea*.

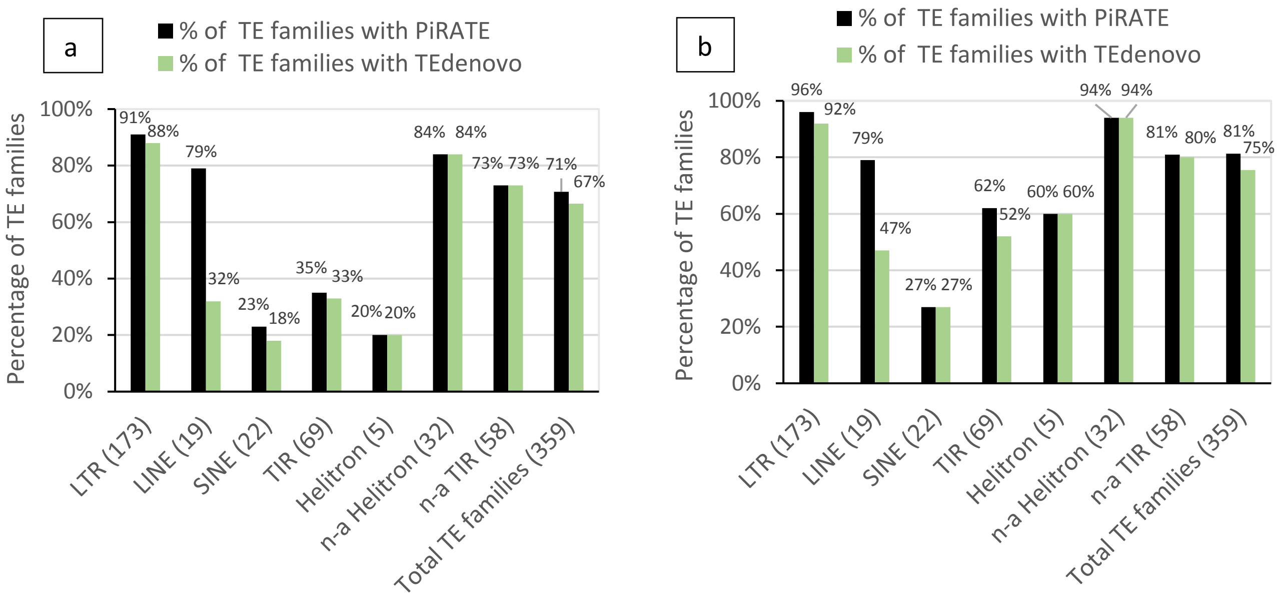

Figure S2: Comparison of the detection step of PiRATE and TEdenovo by calculating the percentage of detected TE families for each TE order of *Arabidopsis thaliana* with: a) a complete length (coverage score ≥ 70%), b) a complete and a partial length (coverage score ≥ 40%). The x-axis indicates the number of TE families for each order; “n-a” means non-autonomous.

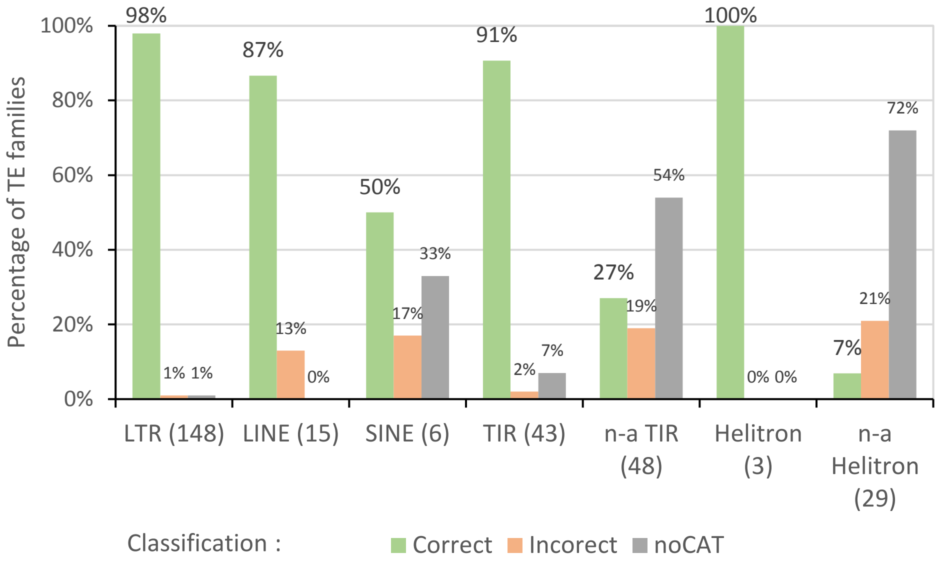

Figure S3: Evaluation of the classification step of PiRATE. Percentage of detected families that were correctly classified, incorrectly classified or classified as uncategorized for each TE order of *Arabidopsis thaliana*. The classification step of PiRATE was able to correctly classify 75% of the detected TE families in *A. thaliania*. The x-axis indicates the number of TE families for each order; "n-a" means non-autonomous.

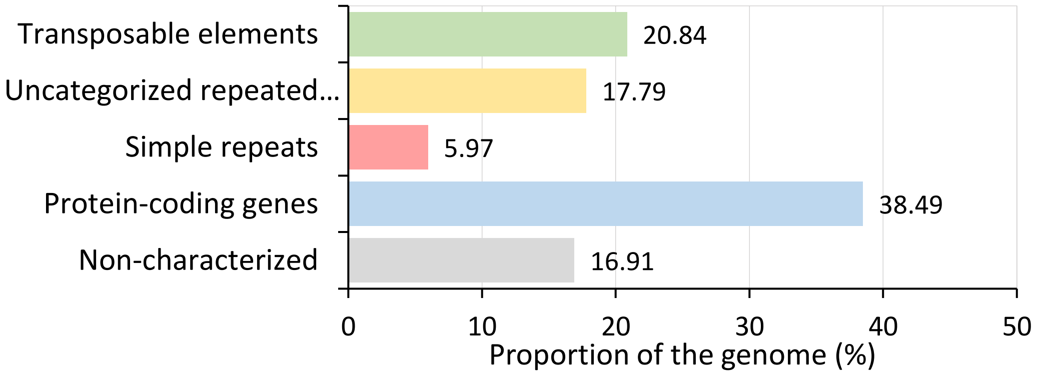

Figure S4: Genome composition of *Tisochrysis lutea*. Proportion of the protein-coding genes, transposable elements, simple repeats, uncategorized repeated elements and uncharacterized sequences.

Method S1: Annotation of the potential autonomous TEs in the genome of *Tisochrysis lutea.*

Using the annotation file obtained with TEannot from the "potential autonomous TEs" library, we established several rules to select potential autonomous TEs depending on the superfamily. First, we established a minimum length depending on the TE order. For example, a minimum length of 4000 bp for the annotated LTR/Copia elements. We also established a "length threshold" value by dividing the "reference TE length" by the "annotate TE length". If this threshold value is 1, a given annotate sequence and its referent sequence have the same length. We choose a minimum and a maximum "length threshold" value for each TE order. We selected the annotated sequences with the highest and lowest threshold value and checked for the presence of conserved domain(s) with Pfam or Blastx. If no domain(s) were found, the value of the threshold was decreased or increased respectively until annotated sequences bearing conserved domain(s) were detected. In addition, we established a minimum percentage of identity for the TIR elements. With a manual check, the most suitable value was 90%. The overview of the applied rules is described in Table S2. Annotated sequences had to meet the required minimum length, minimum % of identity and length thresholds in order not to be excluded.

Table S2: Rules established to annotate potentially autonomous TEs in the genome of *Tisochrysis lutea.*

	**Class I TEs**			**Class II TEs**			
	LTR/Copia	LTR/Gypsy	LINE/L1	TIR/hAT	TIR/Mariner	TIR/PiggyBac	TIR/Harbinger
**Minimum length (bp)**	4000	4000	2500	1300	1000	1000	1000
**Minimum % of identity**	60 (default)	60	60	90	90	90	90
**Minimum length threshold**	/	0.97	0.78	0.98	0.995	0.997	0.93
**Maximum length threshold**	/	1.84	1.2	1.03	1.005	1.003	1.36

Method S2: Annotation of TEs and repeated elements in the genome of *Tisochrysis lutea.*

From the annotations file obtained with TEannot from the "total TE library", we also established several rules to select the potentially autonomous TEs and non-autonomous TEs. We used the same method as that used in Method S1. We established a minimum length of 200 bp to be able to annotate potential TE fossils. We established minimum and a maximum "length threshold" values of 0.5 and 1.5, respectively. We also chose a minimum percentage of identity of 90% for the TIR elements. Annotated sequences had to meet the minimum length, minimum % of identity and length thresholds in order not to be excluded. The overview of the applied rules is given in Table S3. Finally, to measure the proportion of the uncategorized repeated elements of the *T. lutea* genome, we used the annotations file obtained with TEannot from the "repeated elements library". We established a minimum length of 200 bp. The proportion of every annotated repeated element in the genome of *T. lutea* was obtained. We subtracted this proportion from the "total TEs" proportion to obtain an estimation of the uncategorized repeats.

Table S3: Rules established to annotate the TE content in the *Tisochrysis lutea* genome

	**Class I TEs**						**Class II TEs**				
	LTR/Copia	LTR/Gypsy	LINE/L1	TRIM	LARD	SINE	TIR/hAT	TIR/Mariner	TIR/PiggyBac	TIR/Harbinger	MITE
**Minimum length (bp)**	200	200	200	200	200	200	200	200	200	200	200
**Minimum % of identity**	60	60	60	60	60	60	90	90	90	90	90
**Minimum length threshold**	0.5	0.5	0.5	0.5	0.5	0.5	0.5	0.5	0.5	0.5	0.5
**Maximum length threshold**	1.5	1.5	1.5	1.5	1.5	1.5	1.5	1.5	1.5	1.5	1.5

Method S3: Contribution of each TE detection approach, depending on the input data.

The detection step (Fig. 1) of PiRATE was launched with raw *Tisochrysis lutea* Illumina data and either the previous draft version of the *T. lutea* genome or the new *T. lutea* genome. For both these cases the detected sequences were compared with PASTEC to the 240 reference sequences representing the 174 potentially autonomous TE families that we found in the *T. lutea* genome (Results and Discussion, 2.4.1). For both cases, we selected each detected sequence matching with a *T. lutea* TE family. For each detected TE family, we selected the detected sequences with the highest percentage of coverage. We normalized the percentage of coverage of the detected sequences obtained from the draft genome assembly and raw Illumina data with the percentages of coverage of the corresponding detected sequences obtained with the new genome and raw Illumina data. We counted the number of TE families that were detected from the draft genome assembly and the raw Illumina data (having a normalized percentage of coverage of at least 40%). We estimated the contribution of each TE detection approach, depending on the input data. For each detection approach, we counted the number of TE families of *T. lutea* detected with the largest length (highest percentage of coverage compares to reference TE sequences) and divided this number by the total of TE families detected. This provided an estimation of the contribution of each TE detection approach depending on the input data (Main manuscript Fig. 3).
