# Supplementary figures and images for: A transposable element annotation pipeline and expression analysis reveal potentially active elements in the microalga Tisochrysis lutea
Source: BMC Genomics. 2018 May 22;19:378. doi: 10.1186/s12864-018-4763-1 (PMC5963040; doi:10.1186/s12864-018-4763-1)

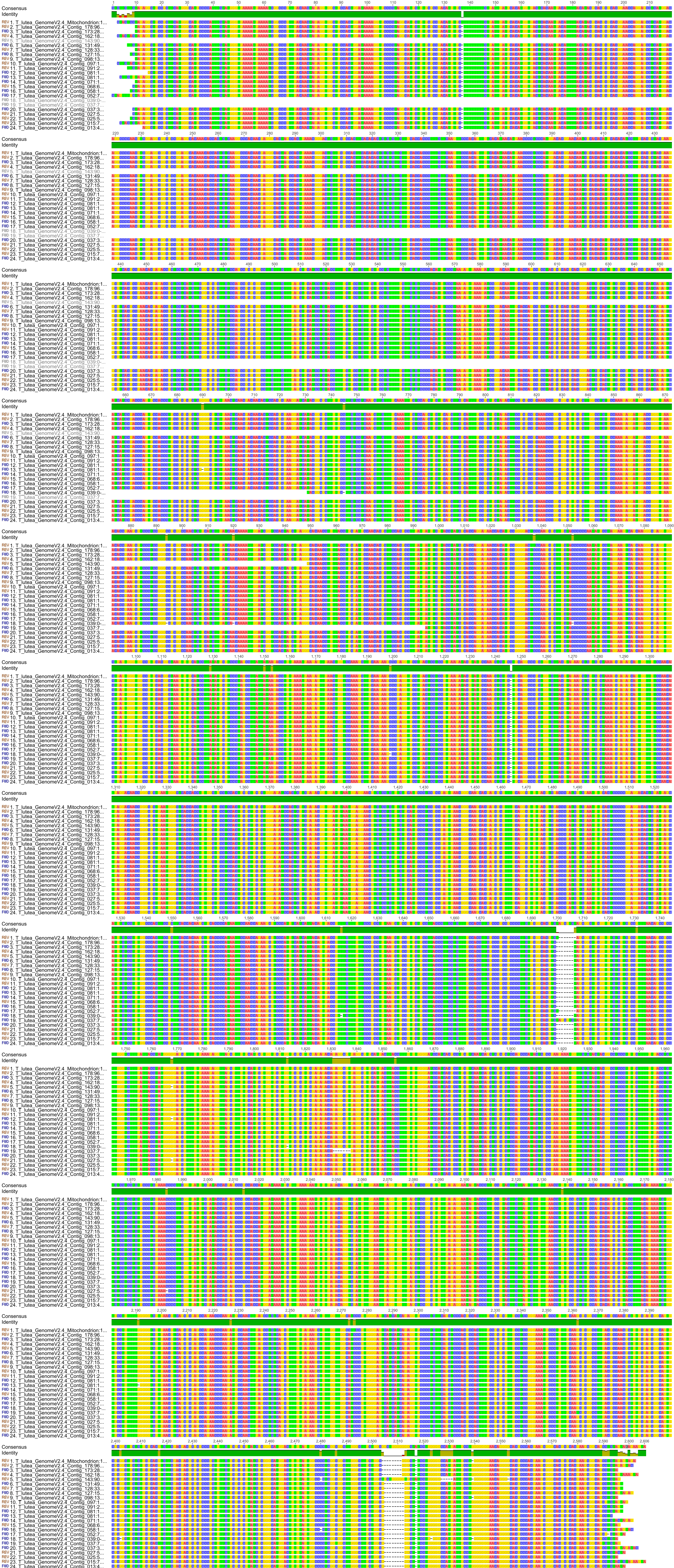

Supplement: Supplementary file 3 — Sequences alignment of TE copies of the TIR/Mariner Luffy family. This file contains the sequence alignment of the copies belonging to the TIR/Mariner Luffy described in the genome of Tisochrysis lutea. (PDF 17427 kb) [file 12864_2018_4763_MOESM3_ESM.pdf]
