# Supplementary material for: A transposable element annotation pipeline and expression analysis reveal potentially active elements in the microalga Tisochrysis lutea
Source: BMC Genomics. 2018 May 22;19:378. doi: 10.1186/s12864-018-4763-1 (PMC5963040; doi:10.1186/s12864-018-4763-1)
Supplement: Supplementary file 4 — Sequences alignment of the peptides matching on the predicted TE proteins. This file contains the alignment of the peptides matching on the predicted proteins of the TIR/Mariner Luffy and the TIR/hAT Ace. (PDF 996 kb) [file 12864_2018_4763_MOESM4_ESM.pdf]

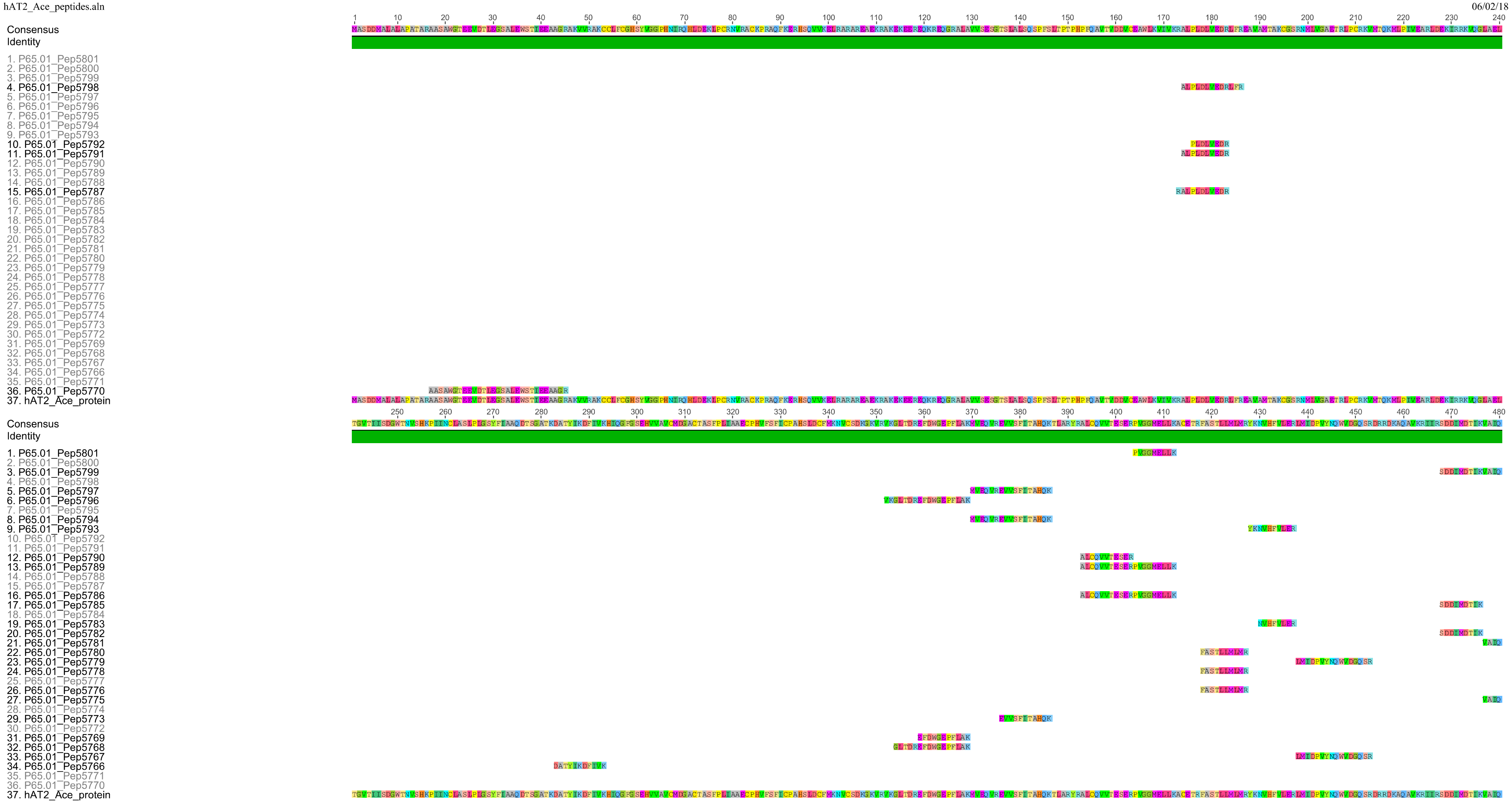
Consensus
Identity
1. P65.01_Pep5801
2. P65.01_Pep5800
3. P65.01_Pep5799
4. P65.01_Pep5798
5. P65.01_Pep5797
6. P65.01_Pep5796
7. P65.01_Pep5795
8. P65.01_Pep5794
9. P65.01_Pep5793
10. P65.01_Pep5792
11. P65.01_Pep5791
12. P65.01_Pep5790
13. P65.01_Pep5789
14. P65.01_Pep5788
15. P65.01_Pep5787
16. P65.01_Pep5786
17. P65.01_Pep5785
18. P65.01_Pep5784
19. P65.01_Pep5783
20. P65.01_Pep5782
21. P65.01_Pep5781
22. P65.01_Pep5780
23. P65.01_Pep5779
24. P65.01_Pep5778
25. P65.01_Pep5777
26. P65.01_Pep5776
27. P65.01_Pep5775
28. P65.01_Pep5774
29. P65.01_Pep5773
30. P65.01_Pep5772
31. P65.01_Pep5769
32. P65.01_Pep5768
33. P65.01_Pep5767
34. P65.01_Pep5766
35. P65.01_Pep5771
36. P65.01_Pep5770
37. hAT2_Ace_protein

	490 … 660
Consensus	IMEPVYRLLRLCDGKLGANLGKVYGYMLQIDTHLRTGIAALDARKRHKIHELLMARWEYFHAPVMTAAYCLEPEYCRRKFSSQELKELKACLKQMATRDHSYPDILADLADFQEACTSGLFDLTDDVAFSNRAKTMASYKWANVYLSHWPHLKWAACRLLALSCSASGCERSWSVEDWIH
Identity	
1. P65.01_Pep5801	
2. P65.01_Pep5800	GLFDLTDDVAFSNR
3. P65.01_Pep5799	IMEPVYR
4. P65.01_Pep5798	
5. P65.01_Pep5797	
6. P65.01_Pep5796	
7. P65.01_Pep5795	VYGYMLQIDTHLR
8. P65.01_Pep5794	
9. P65.01_Pep5793	
10. P65.01_Pep5792	
11. P65.01_Pep5791	
12. P65.01_Pep5790	
13. P65.01_Pep5789	
14. P65.01_Pep5788	DHSYPDILADLADFQEA
15. P65.01_Pep5787	
16. P65.01_Pep5786	
17. P65.01_Pep5785	
18. P65.01_Pep5784	TGIAALDAR
19. P65.01_Pep5783	
20. P65.01_Pep5782	
21. P65.01_Pep5781	IMEPVYR
22. P65.01_Pep5780	
23. P65.01_Pep5779	
24. P65.01_Pep5778	
25. P65.01_Pep5777	SWSVEDWIH
26. P65.01_Pep5776	
27. P65.01_Pep5775	IMEPVYR
28. P65.01_Pep5774	LLALSCSASGCER
29. P65.01_Pep5773	
30. P65.01_Pep5772	
31. P65.01_Pep5769	
32. P65.01_Pep5768	
33. P65.01_Pep5767	
34. P65.01_Pep5766	
35. P65.01_Pep5771	DHSYPDILADLADFQEACTSGLFDLTDDVAFSNR
36. P65.01_Pep5770	
37. hAT2_Ace_protein	IMEPVYRLLRLCDGKLGANLGKVYGYMLQIDTHLRTGIAALDARKRHKIHELLMARWEYFHAPVMTAAYCLEPEYCRRKFSSQELKELKACLKQMATRDHSYPDILADLADFQEACTSGLFDLTDDVAFSNRAKTMASYKWANVYLSHWPHLKWAACRLLALSCSASGCERSWSVEDWIH

```
                           1        10        20        30        40        50        60        70        80        90
Consensus                  MMTWDASGWNYGVQLVVKRKWATGDMLIECPPGECTFDDRVDVTRLQTRSCLPVHSAEGMQTEETFLHYIHRLDEWISQRSAVEAATGGEPIQ
Identity
1. P942.01_Pep26259
2. P942.01_Pep26260
3. P942.01_Pep26262
4. P942.01_Pep26263
5. P942.01_Pep26265                                                                                        SAVEAATGGEPIQ
6. P942.01_Pep26266
7. Mariner3_Luffy_protein  MMTWDASGWNYGVQLVVKRKWATGDMLIECPPGECTFDDRVDVTRLQTRSCLPVHSAEGMQTEETFLHYIHRLDEWISQRSAVEAATGGEPIQ
                                 100       110       120       130       140       150       160       170       180
Consensus                  RPVVLMLDNHCSRFSDELLRKTSGPAAELGIRIFTEESGTSGFLQALDQFNSSFHRSYNKARDAYKDAHLAVHGHPLSHLALADFLGILGGSK
Identity
1. P942.01_Pep26259
2. P942.01_Pep26260
3. P942.01_Pep26262                                        IFTEESGTSGFLQALDQFNSSFHR
4. P942.01_Pep26263
5. P942.01_Pep26265        R
6. P942.01_Pep26266                     FSDELLR
7. Mariner3_Luffy_protein  RPVVLMLDNHCSRFSDELLRKTSGPAAELGIRIFTEESGTSGFLQALDQFNSSFHRSYNKARDAYKDAHLAVHGHPLSHLALADFLGILGGSK
                              190       200       210       220       230       240       250       260       270
Consensus                  RLGVPGMWFSWCDRYDIIKAWKRVGIAGNRLCPQLVDRSNFVDQDAELAAARRDASVSSPGPSSLAEAKRTPPGLRRGSLAAANAKLQQLEQF
Identity
1. P942.01_Pep26259
2. P942.01_Pep26260                                              SNFVDQDAELAAAR
3. P942.01_Pep26262
4. P942.01_Pep26263                                                             DASVSSPGPSSLAEAK
5. P942.01_Pep26265
6. P942.01_Pep26266
7. Mariner3_Luffy_protein  RLGVPGMWFSWCDRYDIIKAWKRVGIAGNRLCPQLVDRSNFVDQDAELAAARRDASVSSPGPSSLAEAKRTPPGLRRGSLAAANAKLQQLEQF
                           280      290       300       310       320       330       340       350       360       370
Consensus                  AKKLEEKAQAPYDPSRGVLSLSSDAPSARQGGTGSDDDSDAADGDSASPVRARRRLVQLSGSFTLRDMWGEKQKRQQEAAKVEDAAQRKKAAR
Identity
1. P942.01_Pep26259                        GVLSLSSDAPSAR
2. P942.01_Pep26260
3. P942.01_Pep26262
4. P942.01_Pep26263
5. P942.01_Pep26265
6. P942.01_Pep26266
7. Mariner3_Luffy_protein  AKKLEEKAQAPYDPSRGVLSLSSDAPSARQGGTGSDDDSDAADGDSASPVRARRRLVQLSGSFTLRDMWGEKQKRQQEAAKVEDAAQRKKAAR
                                  380       390       400       410       420       430       440       450       460 463
Consensus                  LEKRAEDEERSRVRRDAFVACKDVCMCAIVPCPWEGFMLCDNCGNLKKGWCKKRECVKWRKSPVSVASALVVGLESGVCASGAASDVGACP
Identity
1. P942.01_Pep26259
2. P942.01_Pep26260
3. P942.01_Pep26262
4. P942.01_Pep26263
5. P942.01_Pep26265
6. P942.01_Pep26266
7. Mariner3_Luffy_protein  LEKRAEDEERSRVRRDAFVACKDVCMCAIVPCPWEGFMLCDNCGNLKKGWCKKRECVKWRKSPVSVASALVVGLESGVCASGAASDVGACP
```
